# Outcomes of Volumetric-Modulated Arc Therapy for Refractory, Corticosteroid-Resistant Orbital Inflammatory Diseases

**DOI:** 10.1016/j.adro.2025.101811

**Published:** 2025-05-17

**Authors:** Yan Ma, Hai-Yang Chen, Fu-Jing Huang, Xiao-Lin Pang, Jian Zheng, Fang He

**Affiliations:** a Department of Radiation Oncology, The Sixth Affiliated Hospital, Sun Yat-sen University, Guangzhou, China; b Biomedical Innovation Center, The Sixth Affiliated Hospital, Sun Yat-sen University, Guangzhou, China; c Department of Radiation Oncology, Affiliated Hospital, Shandong University of Traditional Chinese Medicine, Jinan, China

## Abstract

**Purpose:**

This study aimed to evaluate the efficacy and safety of volumetric-modulated arc therapy (VMAT) in treating corticosteroid-resistant orbital inflammatory diseases, with a focus on radiation therapy (RT) plan design, clinical outcomes, and the incidence of treatment-related side effects.

**Methods and Materials:**

A retrospective analysis was conducted on 57 patients with refractory, corticosteroid-resistant orbital inflammatory diseases treated with orbital VMAT from November 2019 to July 2022. The primary endpoint was the reduction or cessation of corticosteroid use following RT, with secondary endpoints, including improvements in ocular clinical symptoms (diplopia, proptosis, visual acuity, and extraocular movement) and long-term side effects.

**Results:**

The median target dose was 20 Gy, with an average lens irradiation dose of 5.4 Gy. Initially, all 57 patients received corticosteroids. After a median follow-up of 27.5 months, 89.5% (51 of 57) of patients responded positively to RT; in particular, 56.1% (32 of 57) completely tapered off corticosteroids, whereas 33.3% (19 of 57) reduced their dosage. Symptomatic improvements were observed in diplopia (67.3%), proptosis (64.7%), visual acuity (56.1%), and extraocular movements (65.9%). Regarding the long-term side effects of RT, incidences of dry eye syndrome and lens opacities were reported at 3.5% and 1.8%, respectively.

**Conclusions:**

Orbital VMAT is an effective treatment for refractory, corticosteroid-resistant orbital inflammatory diseases, reducing corticosteroid use and improving ocular symptoms with minimal side effects. Further prospective clinical trials are warranted to validate more appropriate VMAT doses and planning models, enhancing treatment outcomes without increasing RT side effects.

## Introduction

Orbital inflammatory diseases (OID) encompass a broad spectrum of immune-associated disorders that can be categorized into specific and nonspecific types. Specific OIDs are associated with identifiable systemic diseases or underlying causes, including Graves’ ophthalmopathy (GO), immunoglobulin G4-related ophthalmic disease, sarcoidosis, and anti-neutrophil cytoplasmic antibody-associated vasculitis. In contrast, nonspecific OID refers to orbital pseudotumor (OP), also known as idiopathic orbital inflammation, where no identifiable systemic disease or specific cause can be determined.^1^, ^2^, ^3^, ^4^ According to recent population-based studies, GO remains the most prevalent orbital inflammatory disorder, with an estimated annual incidence of 19 per 100,000 population, accounting for over 80% of orbital inflammatory cases.^5^ Common symptoms across these conditions include proptosis, pain, tearing, impaired extraocular mobility, diplopia, periorbital edema, visual impairment, and, in severe cases, blindness*.* Because of the heterogeneous nature of OID, treatment approaches vary, including observation, corticosteroids, immunosuppressive medications, orbital radiation therapy (RT), and surgery.^2^^,^^6^ However, no universally established standard treatment protocol exists because of the limited number of large-scale randomized controlled trials.

Corticosteroids remain the first line of therapy for most OID cases, including nonspecific OID. Still, their long-term or high-dose use may result in significant complications such as hyperglycemia, hypertension, immune suppression, osteoporosis, weight gain, and mood alterations.^7^^,^^8^ Although approximately 80% of patients show improvement, the relapse can be as high as 50%, and corticosteroids may be unsuitable for some individuals because of adverse reactions or contraindications.^9^

Orbital RT has emerged as an alternative, particularly for patients who are unresponsive to or intolerant of corticosteroids. While some studies report response rates between 50% and 97% based on different evaluation criteria, with a median recurrence rate of 10%, and combining RT with corticosteroids often yields superior outcomes,^8^^,^^10^, ^11^, ^12^, ^13^, ^14^ conflicting results from randomized trials suggest that RT may not benefit all patients, which employed a 20∼29-Gy dose of 3-dimensional conformal RT.^15^^,^^16^ Compared to conventional 3-dimensional conformal RT, volumetric-modulated arc therapy (VMAT) offers better dose conformity and organs at risk (OARs) sparing, reducing exposure to the lens and lacrimal glands.^17^ This minimizes side effects such as dry eye syndrome and lens opacities, making VMAT a preferred choice.

This study retrospectively evaluated the efficacy and safety of VMAT in treating refractory, corticosteroid-resistant OID, aiming to analyze the therapeutic efficacy and side effects of RT for these patients.

## Methods and Materials

### Patient selection

The refractory, corticosteroid-resistant OID patients receiving VMAT between November 2019 and July 2022 in our department were retrospectively recruited. The inclusion criteria were as follows: (1) ages 18 to 75 years; (2) moderate-to-severe cases based on clinical severity (proptosis >2 mm above the normal range, diplopia, vision impairment, or a clinical activity score ≥ 4); (3) no prior orbital RT; (4) inability to tolerate or failure to respond to corticosteroids after an optimal trial (defined as a dosage of at least 1 mg/kg/d of oral prednisone or an equivalent dose of another corticosteroid for a minimum of 2 weeks); (5) active inflammatory stage as determined by clinical signs (proptosis, pain, diplopia, and redness) and confirmed by imaging studies (orbital tissue enhancement and edema); and (6) exclusion of cases where chronic fibrosis predominated. Of the initial 60 patients identified, 2 patients with GO were excluded because of an inability to complete the planned course of RT, and 1 patient with GO was excluded because of a short follow-up time of only 2 months. This study was approved by the local institutional ethics committee (approval number: 2023ZSLYEC-084). All patients provided informed consent.

### Target delineation and plan design

Patients were positioned supine and immobilized using a thermoplastic mask system, using the Philips Brilliance Big Bore computed tomography scanner (Philips Medical Systems) following established protocols, with a 3-mm slice thickness spanning from the calvaria to the C2 level. In addition, axial 3.0-mm-thick magnetic resonance images were acquired within the region of interest to facilitate precise target volume delineation.

Clinical target volume (CTV) was defined to encompass the inflamed orbital tissues, meticulously excluding structures such as the lenses, eyes, and anterior chambers, based on symptoms and imaging modalities such as computed tomography or magnetic resonance images. Experienced radiation oncologists meticulously contoured OARs, including lenses, eyeballs, optic nerves, chiasm, and bilateral hippocampi, on the computed tomography data set. Subsequently, a 5∼10-mm expansion was applied to the left and right lenses to generate the lens avoidance (LA) region. Planning target volume (PTV) was then outlined with a 3-mm margin from CTV, excluding the LA region.

The total prescription dose for the PTV was 20∼30 Gy at 2 Gy per once-daily fraction, tailored to individual patient characteristics, extent of inflammation, and severity of symptoms. The planning objective was to ensure that 95% of the PTV received the prescription dose while sparing OARs as low as possible.

For treatment delivery, the Elekta Synergy accelerator equipped with 80 multileaf collimators and the Monaco treatment planning system (Version 5.11.03) were used. All patients underwent treatment with VMAT using 6-MV photon fields. The treatment optimization process employed constraints based on biological cost functions (ie, serial or parallel complication model for OARs and Poisson cell kill function for the PTV).

### Plan evaluation

A quantitative evaluation of the VMAT plans was conducted using a standard dose-volume histogram. For PTV, the following parameters were assessed: volume receiving the prescribed dose was reported as the target coverage, and D_98%_ and D_2%_ (the dose received by 98% and 2% of the PTV volume) were defined as the minimum dose and maximum dose of the PTV.^18^ For OARs, the evaluation included: the maximum dose (denoted as D_max_), defined as the dose to the hottest 0.03-cc volume of the OARs, and D_100%_ (the dose received by 100% of the OAR volume).

### Follow-up and outcomes

The study implemented a structured follow-up protocol to assess the outcomes of RT in patients with OID. Toxicities were graded according to the National Cancer Institute Common Terminology Criteria for Adverse Events version 5.0. Here are the key aspects of the follow-up process and measured outcomes.

#### Primary endpoint: response to RT

The primary endpoint was the response to RT. This was defined as the ability of patients to discontinue or reduce their corticosteroid usage at 6 months post-RT completion without experiencing further flares beyond 6 months. A “flare” was defined as any need for additional intervention, such as increased corticosteroid dosage or surgery, because of increased symptoms occurring after 6 months from RT completion.^19^

#### Descriptive statistics

Ophthalmologic evaluations were conducted at baseline (pre-RT) and during follow-up visits at 1, 3, 6, and 12 months post-RT, with subsequent evaluations at 6-month intervals thereafter. All evaluations were performed by an experienced ophthalmologist, with the following parameters assessed.

• Diplopia was evaluated using the Gorman scale, which categorizes the severity of diplopia.
• Proptosis was measured using Hertel exophthalmometry, with changes of ≥2 mm considered clinically significant.
• Visual acuity was assessed using a Snellen chart and converted to LogMAR for statistical comparison.
• Extraocular movements were evaluated by assessing the movements of the extraocular muscles in all directions of gaze.

#### Long-term side effects of RT

The study focused on dry eye syndrome and lens opacities. Dry eye syndrome was assessed by ophthalmologists through Schirmer’s test and patient-reported symptoms. Lens opacities were diagnosed via slit-lamp examination. The evaluation focused on measuring the occurrence and progression of these effects starting from 6 months post-RT completion and onward. In addition, a dilated fundus examination was performed at each follow-up visit to check for potential signs of radiation retinopathy, including microaneurysms, cotton wool spots, and retinal hemorrhages.

### Statistical methods

Statistical analysis and tabulations were conducted using JMP software (SAS Institute). The Kaplan–Meier product limit method was employed to provide an estimate of local control.

## Results

### Patient characteristics

The study population comprised a total of 57 enrolled patients, categorized into different subgroups based on their underlying condition: 33 patients with GO, 21 patients with OP, and 3 patients with immunoglobulin G4-related ophthalmic disease. Table 1 provides a summary of the main patient and tumor characteristics.

**Table 1:** Patient and disease characteristics

	Specific OID		Nonspecific OID
Characteristic	GO (n = 33)	IgG4-ROD (n = 3)	OP (n = 21)
Females/males, n	16 (48.5)/17 (51.5)	1 (33.3)/2 (66.7)	11 (52.4)/10 (47.6)
Age (y), median (range)	45 (27-63)	49 (34-51)	43 (20-58)
CTV volume, cm^3^, median (range)	69.26 (25.22-76.92)	62.58 (32.12-66.65)	32.61 (22.91-64.87)
Location	Left eye: 0	Left eye: 1	Left eye: 5
	Right eye: 2	Right eye: 0	Right eye: 11
	Both eyes: 31	Both eyes: 2	Both eyes: 5
Duration of symptoms (mo)	7 (1-21)	3 (2-9)	4 (1-15)
Duration of steroid treatment (mo)	6 (1-15)	3 (3-6)	4 (2-9)
Optic neuropathy	5 (13.9)	0	0
EUGOGO Severity Score	Mild: 0 Moderate-to-severe: 27 Sight-threatening: 9	-	-
Smokers	20	1	7
Diabetes	5	0	1

*Abbreviations:* CTV = clinical target volume; EUGOGO = European Group on Graves' Orbitopathy; GO = Graves’ ophthalmopathy; IgG4-ROD = immunoglobulin G4-related ophthalmic disease; OID = orbital inflammatory disease; OP = orbital pseudotumor.

In the GO subgroup, among the 33 patients, the cohort comprised 16 females (48.5%) and 17 males (51.5%). The median age was 45 years, ranging from 27 to 63 years. The median CTV was recorded at 69.26 cm^3^, with a range spanning from 25.22 to 76.92 cm^3^. Bilateral manifestation of the condition was predominant, observed in 31 patients, whereas unilateral affliction was comparatively rare, noted in 2 cases affecting the right eye.

In the OP subgroup, there was a nearly balanced gender distribution, with 11 females (52.4%) and 10 males (47.6%). The median age was slightly lower compared with the GO subgroup, at 43 years, ranging from 20 to 58 years. The median CTV was 32.61 cm^3^, varying between 22.91 and 64.87 cm^3^. Unilateral conditions were more prevalent in the OP subgroup, with 11 patients experiencing right-eye involvement and 5 patients experiencing left-eye involvement. Bilateral affliction was confirmed in 5 cases.

In the immunoglobulin G4-related ophthalmic disease subgroup, only 3 patients were included, with 1 female (33.3%) and 2 males (66.7%). The median age in this subgroup was 49 years, within a narrower range of 34 to 51 years. The median CTV was noted as 62.58 cm^3^, spanning from 32.12 to 66.65 cm^3^. Bilateral disease was present in 2 patients, with 1 patient having left-eye involvement and none with right-eye involvement.

### Target delineation, plan design, and radiation dose analysis

**Figure 1:**
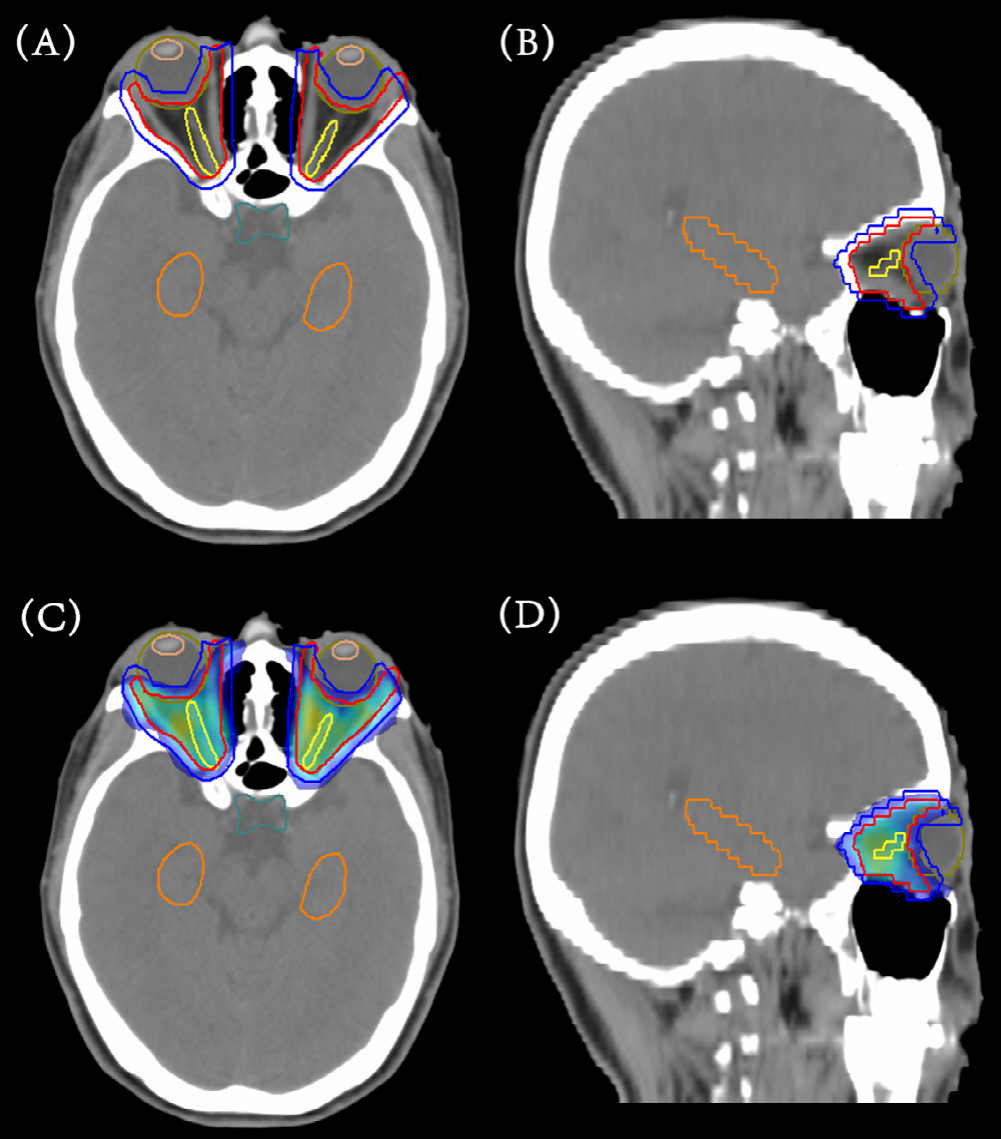
Representative images of a volumetric-modulated arc therapy plan for Graves’ ophthalmopathy. (A) Axial computed tomography (CT) image with clinical target volume delineated in red, planning target volume delineated in blue, and organs at risk. (B) Sagittal CT image with clinical target volume, planning target volume, and OARs. (C) Axial CT image with prescription dose distribution. (D) Sagittal CT image with prescription dose distribution.

Figure 1A, B illustrates the delineation of the CTV for 1 patient, which encompasses the extraocular muscles delineated in red. This volume represents the area requiring treatment to address inflammation and associated symptoms. In addition, Fig. 1C, D depicts the typical dose distribution of a VMAT plan for this patient. A low-dose zone around the lenses, referred to as the LA region, is also shown in Fig. 1. This area is spared from high doses of radiation to prevent complications such as dry eye syndrome and lens opacities.

The plan optimization protocol, detailed in Table 2 (eg, 30 Gy over 15 fractions), incorporates stringent dose limitations for OARs, with a special emphasis on the lenses. Contrary to conventional cancer treatment planning, where the high-dose limit for the PTV is typically restricted to 110% of the prescribed dose, covering less than 5% of the PTV, our modified threshold permits up to 125% of the prescribed dose, restricted to less than 2% of the PTV. This adjustment reflects a strategic shift in dose distribution, aligning with the nuanced requirements of our specific treatment objectives: achieving a steep dose fall-off gradient between the PTV and lenses, and ensuring comprehensive coverage of the prescribed dose for the PTV while restricting the dose to the lenses to a maximum of 6 Gy. In addition, in consideration of hippocampal memory protection, dose constraints were set as follows: D_max_ ≤ 16 Gy and D_100%_ ≤ 9 Gy.^20^

**Table 2:** Radiation therapy planning optimization protocol (eg, 30 Gy/15 f)

Name of structure	Dosimetric parameter	Per protocol	Notes
PTV_30	D_2%_	≤37.5 Gy*	Dose to hottest 2% of PTV_30
	D_98%_	≥25 Gy	Dose to 98% of PTV_30
	V_30 Gy_	≥95 %	Volume receiving prescription dose of 30 Gy
Lens (left side)†	D_max_	≤6 Gy	Dose to hottest 0.03-cc volume of left lens
Lens (right side)†	D_max_	≤6 Gy	Dose to hottest 0.03-cc volume of right lens
Eyeball (left side)	D_max_	≤37.5 Gy*	Dose to hottest 0.03-cc volume of left eyeball
	D_mean_	≤25 Gy	Mean dose to left eyeball
Eyeball (right side)	D_max_	≤37.5 Gy*	Dose to hottest 0.03-cc volume of right eyeball
	D_mean_	≤25 Gy	Mean dose to right eyeball
Optic nerve (left side)	D_max_	≤37.5 Gy*	Dose to hottest 0.03-cc volume of left optic nerve
Optic nerve (right side)	D_max_	≤37.5 Gy*	Dose to hottest 0.03-cc volume of right optic nerve
Hippocampi (left side)	D_max_	≤16 Gy	Dose to hottest 0.03-cc volume of left hippocampi
	D_100%_	≤9 Gy	Dose to 100% of left hippocampi
Hippocampi (right side)	D_max_	≤16 Gy	Dose to hottest 0.03-cc volume of right hippocampi
	D_100%_	≤9 Gy	Dose to 100% of right hippocampi

*Abbreviations:* cc = cubic centimeters; D = dose; PTV = planning target volume.

⁎ 37.5 Gy is 125% of the prescription dose.

† There are no specified dose parameters for the lens avoidance region because this region represents a transition region for dosing.

Table 3 provides the detailed dosimetry statistics on target coverage and OARs. The average coverage of the PTV reached 95.8% with a V_125%_ value of 1.3%. In addition, D_max_ of the lenses was recorded at 5.4 Gy. For the left and right bilateral hippocampi, the D_max_ values were 9.6 and 10.9 Gy, respectively, whereas the D_100%_ values were 2.3 and 3.4 Gy, respectively. Those results indicate strict compliance with the established protocol outlined in Table 2. Such dosimetry data are crucial for assessing the balance between effective tumor control and minimizing the risk of radiation-induced damage to OARs.

**Table 3:** Target coverage and organs at risk sparing statistical results

Structures	Dosimetric parameter	Value (range)
PTV__P_	V_P_	95.8% (93.2%-97.1%)
	V_125%_	1.3% (0.8%-2.0%)
Lens (left side)	D_max_ (Gy)	5.4 (4.2-5.9)
Lens (right side)	D_max_ (Gy)	5.4 (4.7-5.8)
Eyeball (left side)	D_max_ (Gy)	22.3 (16.2-24.1)
	D_mean_ (Gy)	16.2 (14.3-17.8)
Eyeball (right side)	D_max_ (Gy)	24.4 (20.6-26.9)
	D_mean_ (Gy)	17.6 (15.9-19.2)
Optic chiasm	D_max_ (Gy)	17.9 (15.2-19.7)
Optic nerve (left side)	D_max_ (Gy)	22.7 (20.1-24.9)
Optic nerve (right side)	D_max_ (Gy)	24.4 (22.1-26.8)
Bilateral hippocampi (left side)	D_max_ (Gy)	9.6 (8.2-11.3)
	D_100%_ (Gy)	2.3 (1.8-3.1)
Bilateral hippocampi (right side)	D_max_ (Gy)	10.9 (9.4-12.5)
	D_100%_ (Gy)	3.4 (2.6-4.3)

*Abbreviations:* cc = cubic centimeters; D = dose; PTV = planning target volume; V_P_ = volume receiving the prescribed dose.

### Radiation treatment response and toxicities

The median follow-up duration in the study was 27.5 months, ranging from 6 to 47 months, with 86.0% of the patients having follow-ups ≥12 months.

Further details regarding corticosteroid tapering can be found in Table 4. Corticosteroids were administered as the initial treatment modality to the entire cohort of 57 patients. Of those, 51 patients (51 of 57, 89.5%) responded positively to RT, whereas 6 patients (6 of 57, 10.5%) failed to respond because of uncontrolled symptoms and required further intervention. Specifically, 32 patients (32 of 57, 56.1%) were able to completely taper off corticosteroids because of symptom control, whereas 19 patients (19 of 57, 33.3%) experienced a reduction in corticosteroid dosage among the remaining 25 patients who were unable to fully decrease their corticosteroid dosage. Of the 6 nonresponders (10.5%), treatment failure occurred at a median of 8 months. Subsequent management included: immunosuppressants in 1 patient, orbital decompression surgery in 3 patients, and close observation in 2 patients. Notably, none of the patients received re-irradiation.

**Table 4:** Corticosteroid therapy post-orbital radiation therapy

	Specific OID		Nonspecific OID	
Characteristic	GO (n = 33)	IgG4-ROD (n = 3)	OP (n = 21)	Overall (n = 57)
Able to discontinue corticosteroids	20 (60.6%)	1 (33.3%)	11 (52.4%)	32 (56.1%)
Unable to discontinue corticosteroids	13 (39.4%)	2 (66.7%)	10 (47.6%)	25 (43.9%)
Patients’ decrease in steroid dose in those who were unable to fully reduce their corticosteroid dose	8 (24.2%)	2 (66.7%)	9 (42.9%)	19 (33.3%)
Responded to RT	28 (84.8%)	3 (100.0%)	20 (95.2%)	51 (89.5%)
Failed RT	5 (15.2%)	0 (0.0%)	1 (4.8%)	6 (10.5%)

*Abbreviations:* GO = Graves’ ophthalmopathy; IgG4-ROD = immunoglobulin G4-related ophthalmic disease; OID = orbital inflammatory disease; OP = orbital pseudotumor; RT = radiation therapy.

The symptom changes and long-term side effects before and after RT are presented in Table 5. Notably, symptomatic improvements were observed in diplopia (33 of 49, 67.3%), proptosis (33 of 51, 64.7%), visual acuity (32 of 57, 56.1%), and extraocular movements (29 of 44, 65.9%) among the patients. As for the long-term side effects of RT, the incidences of dry eye syndrome and lens opacities were reported at 3.5% (2 of 57) and 1.8% (1 of 57), respectively.

**Table 5:** Symptom outcomes and long-term side effects post-orbital radiation therapy

	Specific OID		Nonspecific OID	
Outcome	GO (n = 33)	IgG4-ROD (n = 3)	OP (n = 21)	Overall (n = 57)
Symptom outcomes				
Diplopia	29	2	18	49
Improved	18 (62.1%)	1 (50.0%)	14 (77.8%)	33 (67.3%)
Unchanged	7 (24.1%)	1 (50.0%)	3 (16.7%)	11 (22.4%)
Worsened	4 (13.8%)	0 (0.0%)	1 (5.6%)	5 (10.2%)
Proptosis	33	2	16	51
Improved	20 (60.6%)	1 (50.0%)	12 (75.0%)	33 (64.7%)
Unchanged	12 (36.4%)	1 (50.0%)	4 (25.0%)	17 (33.3%)
Worsened	1 (3.0%)	0 (0.0%)	0 (0.0%)	1 (2.0%)
Decreased visual acuity	33	3	21	57
Improved	18 (54.5%)	2 (66.7%)	12 (57.1%)	32 (56.1%)
Unchanged	14 (42.4%)	1 (33.3%)	8 (38.1%)	23 (40.4%)
Worsened	1 (3.0%)	0 (0.0%)	1 (4.8%)	2 (3.5%)
Restricted extraocular movements	25	2	17	44
Improved	16 (64.0%)	1 (50.0%)	12 (70.6%)	29 (65.9%)
Unchanged	8 (32.0%)	1 (50.0%)	4 (23.5%)	13 (29.5%)
Worsened	1 (4.0%)	0 (0.0%)	1 (5.9%)	2 (4.5%)
Long-term side effects	1	0	2	3
Dry eye syndrome	1 (3.0%)	0 (0.0%)	1 (4.8%)	2 (3.5%)
Lens opacities	0 (0.0%)	0 (0.0%)	1 (4.8%)	1 (1.8%)

*Abbreviations:* GO = Graves’ ophthalmopathy; IgG4-ROD = immunoglobulin G4-related ophthalmic disease; OID = orbital inflammatory disease; OP = orbital pseudotumor.

## Discussion

Benign orbital diseases, while not inherently life-threatening, can significantly compromise visual function by affecting critical structures such as the optic nerve, extraocular muscles, or cornea.^3^^,^^4^ The best clinical management of patients diagnosed with OID remains uncertain, despite various treatment modalities that have demonstrated efficacy.^4^ As an alternative option to OID, orbital RT has shown potential in alleviating orbital inflammation and improving visual symptoms in numerous studies; however, its efficacy remains a subject of debate.^15^^,^^16^ To address this ambiguity, our study aimed to assess the effectiveness of VMAT for refractory, corticosteroid-resistant OID patients based on several key outcome measures. Our primary focus was on the patient’s ability to taper corticosteroids without experiencing further exacerbations of orbitopathy symptoms, serving as a crucial indicator of treatment efficacy. In addition, we evaluated changes in specific symptoms such as diplopia, proptosis, visual acuity, and extraocular movements before and after VMAT. Furthermore, we examined long-term side effects, with a particular emphasis on dry eye syndrome and lens opacities. By comprehensively analyzing these parameters, we aimed to provide valuable insights into the overall effectiveness and safety profile of VMAT as a treatment option for OID, ultimately guiding clinical decision-making and improving patient outcomes.

In our study, 89.5% of patients on corticosteroids demonstrated a significant reduction in corticosteroid requirements after orbital VMAT. Among these patients, 56.1% were able to discontinue corticosteroids entirely, and 33.3% experienced reductions in corticosteroid dosage. These findings are consistent with previous studies that have used corticosteroid tapering as a primary measure of RT effectiveness at specific time points.^19^^,^^21^ For the 10.5% of treatment failures, although potential prognostic factors were analyzed, no statistically significant predictors were identified. Moreover, our cohort exhibited a low incidence of RT-related toxicities, with only 3.5% of patients developing dry eye syndrome and 1.8% experiencing lens opacities. Notably, no cases of radiation-induced optic neuropathy or retinopathy were observed. These findings highlight the role of RT in helping stabilize the disease process and minimize the duration of corticosteroid therapy, with an acceptable toxicity profile. Based on our institutional experience and the current study outcomes, the ideal candidates for orbital RT are patients with moderate-to-severe corticosteroid-resistant OID who present with significant clinical symptoms such as diplopia, proptosis, or restricted ocular mobility. These individuals often demonstrate poor tolerance or an inadequate response to systemic corticosteroids and have failed prior immunosuppressive therapies. It should be noted that patients with diabetes may be more susceptible to ocular complications from RT because of pre-existing microvascular damage. Although diabetes should not be regarded as an absolute contraindication, meticulous patient selection and individualized dose planning are essential. Diabetic patients should be closely monitored throughout and after RT for early signs of radiation-induced toxicity.^22^

Previous reports have also established the efficacy of the 20 Gy in 10 fractions as the most efficacious and minimally toxic treatment dose.^10^^,^^19^ However, other reports have suggested that doses less than 20 Gy are sufficient for patients with predominantly soft tissue signs without ocular dysmotility.^17^ Our study, with a prescription of 20∼30 Gy depending on the severity of symptoms, showed a highly favorable outcome.

Orbital RT offers an advantage over surgery by preserving eye structure, potentially leading to a better appearance after treatment. Historically, conventional RT or 3-dimensional conformal RT was commonly employed for OID treatment but lacked dose-modulating techniques. In one approach, protecting the lenses involved forced avoidance, which resulted in inadequate dose coverage of the target area, whereas the other approach aimed at ensuring adequate target coverage but ran the risk of overexposure of the lenses. In a study by Matthiesen et al,^23^ a dose of 20 Gy in 10 fractions was delivered using 2 opposing lateral 6-MV fields. Notably, they reported a 12% incidence of dry eye syndrome. In our investigation, we applied a 5∼10-mm expansion to generate the LA region around the lenses, and we delineated the PTV with a 3-mm margin, excluding the LA region, which effectively reduced the dose to lenses and lacrimal glands. With the advanced orbital VMAT, we achieved a steeper dose gradient between the tumor target and OARs in our study, in which we set strict dose constraints with D_max_ of lenses ≤6 Gy and PTV coverage ≥95%. Our orbital VMAT plans yielded a D_max_ of 5.4 Gy to the lenses and 95.8% average PTV coverage, contributing to low incidences of dry eye syndrome (3.5%) and lens opacities (1.8%), which was consistent with the findings by Li et al.^24^

Thus, our meticulous delineation of PTV, prescription of dose, and dose constraints on OARs proved effective in minimizing radiation-induced side effects while maintaining treatment efficacy. These results underscore the importance of adopting advanced RT techniques like VMAT in the management of OID, highlighting the potential for improved patient outcomes and quality of life. Further studies are warranted to explore the long-term efficacy and safety of this approach, as well as its impact on patient-reported outcomes.

However, there are several limitations to our retrospective analysis: (1) the number of cases is limited to just over 50, suggesting the need for larger sample sizes in future follow-up studies; (2) retrospective studies are inherently prone to selection bias, and unidentified confounding factors may influence the results; (3) the therapeutic dose ranges from 20 to 30 Gy, lacking uniformity, and the optimal RT dose remains uncertain, warranting further investigation through prospective trials; and (4) the relatively short follow-up period may not fully capture the long-term risk of radiation optic neuropathy and retinopathy, which may emerge years after treatment. In addition, the development of lens opacities, a known delayed effect of radiation, may not have been fully captured in our study. This is especially important given that the average age of patients included was <50 years, and younger patients may be at greater risk of developing secondary malignancies over time. Further long-term studies will be necessary to evaluate the true incidence of these late effects. Therefore, prospective studies with well-defined cohort characteristics and outcome definitions are essential to comprehensively assess the safety and efficacy of orbital RT.

## Conclusions

Orbital RT has shown promising results in improving visual symptoms and reducing the dependence on corticosteroids in patients with OID. It provides an alternative treatment option for patients who cannot tolerate or have contraindications to prolonged corticosteroid therapy. The use of RT has demonstrated acceptable side effects, although long-term effects still need to be monitored and evaluated through further follow-up. Larger and well-designed prospective trials are warranted to establish standardized protocols and guidelines for the RT management of OID.

## Disclosures

The authors declare that they have no known competing financial interests or personal relationships that could have appeared to influence the work reported in this paper.
